# Minimal Information for Neural Electromagnetic Ontologies (MINEMO): A standards-compliant method for analysis and integration of event-related potentials (ERP) data

**DOI:** 10.4056/sigs.2025347

**Published:** 2011-11-15

**Authors:** Gwen Frishkoff, Jason Sydes, Kurt Mueller, Robert Frank, Tim Curran, John Connolly, Kerry Kilborn, Dennis Molfese, Charles Perfetti, Allen Malony

**Affiliations:** 1 Department of Psychology & Neuroscience Institute, Georgia State University, Atlanta, GA; 2 NeuroInformatics Center, University of Oregon, Eugene, OR; 3 Neural ElectroMagnetic Ontologies (NEMO) ERP Consortium; 4 Department of Computer & Information Science, University of Oregon, Eugene, OR; 5 Department of Psychology & Neuroscience, University of Colorado, Boulder, CO; 6 Department of Psychology and Linguistics, McMaster University, Ontario, CA; 7 Department of Psychology, Glasgow University, Scotland, UK; 8 Department of Psychology, University of Nebraska-Lincoln, Lincoln, NE; 9 Department of Psychology and Linguistics, University of Pittsburgh, Pittsburgh, PA

**Keywords:** Ontology, database, data sharing, standardization, experiment metadata, neuroscience, electrophysiology, event-related potentials

## Abstract

We present *MINEMO* (Minimal Information for Neural ElectroMagnetic Ontologies), a checklist for the description of event-related potentials (ERP) studies. MINEMO extends MINI (Minimal Information for Neuroscience Investigations)to the ERP domain. Checklist terms are explicated in NEMO, a formal ontology that is designed to support ERP data sharing and integration. MINEMO is also linked to an ERP database and web application (the *NEMO portal*). Users upload their data and enter MINEMO information through the portal. The database then stores these entries in RDF (Resource Description Framework), along with summary metrics, i.e., spatial and temporal metadata. Together these spatial, temporal, and functional metadata provide a complete description of ERP data and the context in which these data were acquired. The RDF files then serve as inputs to ontology-based labeling and meta-analysis. Our ultimate goal is to represent ERPs using a rich semantic structure, so results can be queried at multiple levels, to stimulate novel hypotheses and to promote a high-level, integrative account of ERP results across diverse study methods and paradigms.

## Introduction

Over the last few decades, neuroscience has witnessed an explosion of methods for the measurement of human brain function, including high-density (multi-sensor) event-related potentials (ERPs). In comparison with other techniques, the ERP method has several advantages: it is completely safe and noninvasive, it is inexpensive and portable, and — unlike methods such as functional magnetic resonance imaging (fMRI) — it is a direct measure of neuronal activity. The ERP method also has excellent (millisecond) temporal resolution, which is critical for representation of neural dynamics. Remarkably, despite these many virtues, there are few quantitative comparisons (“meta-analyses”) of ERP results, reflecting the complexity of ERP data and the wide variety of methods that are used to extract and analyze ERP metadata [1-3].

To address this gap, we have gathered an interdisciplinary team of researchers in informatics and human neuroscience to form project NEMO (Neural ElectroMagnetic Ontologies). Our neuroscience experts are internationally known for their ERP studies of language and cognition and have partnered to form a consortium. Consortium members provide ERP datasets and contribute to the design and testing of ERP ontologies and ontology-based methods for meta-analysis [3].

In the present paper, we present a minimal information checklist, called MINEMO (Minimal Information for NEMO). MINEMO specifies the key information that should be provided when an ERP experiment is uploaded to the NEMO database. MINEMO terms are explicated in the NEMO ontology, a formal semantic system that we have created for the ERP domain. We have also developed a web application (the NEMO portal) and database, which are aligned with the MINEMO checklist and ontology. Together, the checklist, ontology, and database are intended to support the first complete, cross-laboratory meta-analysis for the ERP domain.

The rest of this paper is structured as follows. In Section 2, we outline prior work on the development of minimal information (MI) checklists, controlled vocabularies, and formal semantic systems (ontologies). In Section 3, we present the MINEMO checklist. In Section 4 we describe how MINEMO is aligned with the NEMO ontology and how it is linked to the NEMO database and portal. Section 5 provides a brief a summary and describes ongoing and future work.

## Related work

In this section we describe prior work that has informed the development of MINEMO. This work falls into three categories: ***Standardized checklists***, which specify key ("minimal") information for representation of data in a particular domain; (2) ***Controlled vocabularies***, which prescribe standard terms, together with human-readable definitions, for consistent annotation of data; and (3) ***Formal ontologies***, which include defined classes, class hierarchies, relations between classes, and axioms for reasoning over class- and instance-level information.

### Standardized Checklists

The Minimum Information for Biological and Biomedical Investigations (MIBBI) is a pioneering project that aims to coordinate guidelines for reporting of scientific metadata across domains [4]. Central to this effort is the MIBBI portal, a clearinghouse for proposed MI checklists. The motivation for MIBBI is two-fold: (1) to promote the use of standard checklists by various stake-holders (e.g., journals, authors, reviewers, and funders), and (2) to facilitate "harmonization," that is, mapping or integration, of domain-specific guidelines. To the extent that researchers can agree on these guidelines, the MIBBI effort may constitute an important first step towards widespread data sharing within and across biological domains.

One checklist that is available through the MIBBI portal is the Minimal Information for Neuroscience Investigations, or MINI, checklist [5]. MINI specifies guidelines for reporting of electrophysiology experiments and comprises eight sets of fields (i.e., tables): (1) General features of an experiment, (2) Study subject(s), (3) Anatomical location of electrophysiological recording, (4) Experimental task, (5) Experimental stimuli, (6) Behavioral response data, (7) Recording specifications and (8) Electrical (time series) data. MINI is intended to cover a wide range of electrophysiological protocols, but appears best suited for reporting on single-cell recordings, as opposed to far-field recordings, such as EEG and ERPs.

In human neuroscience, Poldrack and associates have proposed a set of standards for reporting of fMRI data, called MIfMRI (see MIBBI portal and Appendix A in Ref [6].). MIfMRI specifies minimal information about human subjects, a useful complement to MINI, and categories such as Task and Behavioral performance, which are available in MINI and can be readily extended to other types of human neuroscience protocols (e.g., ERP experiments). Other categories, such as experimental design, appear more narrowly suited for description for fMRI experiments.

There are several publications on ERP research design, implementation, and reporting of results [7-9], but no minimal information checklists or similar resources for the ERP domain. In 2000, Picton and associates provided a detailed and highly influential set of guidelines [9]. In developing MINEMO, we have taken these guidelines under consideration. At the same time, we have tried to create a usable (i.e., relatively short) checklist, comprising no more than ~60 fields— and no more than ~20 that must be completed before data are uploaded to the NEMO database. In this respect, we follow BrainMap and MIBBI researchers, who have discussed lessons learned in developing metadata tools and resources and then working to secure buy-in from users [4,10]. However good the resource, it is unlikely to find widespread use if it is clunky or time-consuming to use.

### Controlled Vocabularies

For the NEMO project, we need consistent annotation of ERPdata, since we are aiming to conduct cross-lab meta-analysis. MI checklists can promote the use of consistent guidelines for reporting of studydata. However, there is no guarantee that different researchers will use the same terms for data mark-up. For this reason, researchers in several domains have created controlled vocabularies, or lexicons, for data annotation [11]^1^.

In human neuroscience, the BrainMap lexicon has enjoyed widespread use, particularly in connection with their database [10,12]. The BrainMap database is an immense repository, resulting from more than 10 years of work curating results from thousands of functional brain imaging studies. Making such a collection reliably searchable requires consistent and precise naming of study information. To this end, the BrainMap team has created a portal called ‘Sleuth’ that supports controlled entry of metadata. The BrainMap lexicon (aka the ‘Meta-Data Coding Scheme’) covers a range of metadata, including stimuli, tasks (instructions), and protocols for measurement of behavioral and brain responses. In addition to historical (and often idiosyncratic) terms for paradigms, such as the ‘Stroop Task’ or ‘Auditory Oddball Task’, each set of results that is entered in BrainMap is linked to a specific Stimulus, Task (Instructions), and Response category. Recent studies have used data mining to uncover patterns of brain activation across different paradigms that share stimulus, task, and/or response properties, demonstrating the utility of fine-grained, consistent annotation of experiments [13].

### Formal Ontologies

A recent trend in bio- and neuro-informatics is the creation of domain ontologies [14]. Like a controlled vocabulary, an ontology contains semantic categories or classes that refer to well-defined entities (e.g., 'stimulus', 'response'). Each class has a uniform resource identifier, or URI, which is globally unique (e.g.,http://purl.bioontology.org/NEMO/ontology/NEMO.owl#NEMO_4762000), in addition to a human-readable label (e.g., ‘onset_stimulus_presentation’). In addition, ontologies specify the semantic relations between classes (e.g., ‘onset_stimulus_presentation *proper_part_of* some presentation_of_stimulus’). These relations are called object properties and impart much of the power behind ontologies. For example, in NEMO the object property *rostral_to* is transitive and has an inverse property, *caudal_to*. Thus, the assertions '(Electrode) Fz *rostral_to* (electrode) Cz' and '(Electrode) Pz *caudal_to* (electrode) Cz' support the inference that '(Electrode) Fz *rostral_to* (electrode) Pz'. Assertions can be built into the ontology (e.g., as class restrictions). When they are defined as equivalent class statements, they can serve as rules to support classification of instance-level information (e.g., scientific data).

In NEMO, ERP patterns are associated with rules that specify the spatial, temporal, and functional (experimental) properties that are required for an ERP observation to be classified as a particular kind of pattern. In this way, the ontology becomes more than a static resource: it functions as a dynamic tool for interpretation of data in the context of a larger base of knowledge.

NEMO has adopted many of the recommended practices outlined by the OBO Foundry [15], including re-use of existing resources (checklists, ontologies, etc.), modularity or orthogonality, human-readable annotations, and — perhaps most important — use of the Basic Formal Ontology (BFO) as an upper ontology and the Ontology of Biological Investigations (OBI) as a mid-level ontology [15]. In doing so, we have joined a community of researchers who have adopted similar practices in order to facilitate collaborative development and harmonization of neuroscience resources. For example, the Neuroscience Information Framework (NIF) [15-17] is a leading project that aggregates online sources of neuroscience data, including databases, web sites, publications, and XML files, and provides a search interface across these disparate sources. An essential part of this effort is the NIF ontology (NIFSTD [15]; ), which extends the older BirnLex ontology to cover additional domains, such as neurons, genetics, proteomics, and phenotypes. The BirnLex ontology has also given rise to the cognitive paradigms ontology, or cogPO [18]. CogPO is also based on BFO and OBI, and is building a formal ontology that uses the BrainMap Metadata Coding Scheme as a starting point. NEMO has been working closely with cogPO and NIF to coordinate ontology development efforts, particularly in the specification of experiment metadata.

### Minimal information for NEMO (MINEMO)

The MINEMO checklist was intended to augment other NEMO resources that are used to support cross-lab analysis, storage, and integration of ERP data. MINEMO extends MINI [5]to the ERP domain. In doing so, it re-uses (in whole or in part) all but one of the MINI tables ("recording location" is specific to invasive recordings and was replaced by information about EEG sensor layouts). We also made the following changes. First, we split the first table in MINI (General features) into three sets of metadata: Research Lab (PI, PI institution and contact information), Experiment (General Features), and Publication. The remaining tables were amended to reflect the use of human subjects, as well as key recording and analysis methods that are specific to ERP research. The resulting checklist comprises ~70 fields (see Appendix A), enough information — we believe — to obtain a thorough, yet compact summary of ERP datasets. Each checklist item is linked to a key term, which is fully explicated — that is, defined and annotated — within the NEMO ontology. Appendix B provides the NEMO URI for each of the MINEMO key terms.

NEMO consortium members have been very willing to provide the complete set of metadata for each of their datasets. In practice, though, some metadata is harder to locate, particularly for legacy datasets. We therefore decided to specify a subset of MINEMO terms that would be required for the first stage of meta-data entry through the NEMO portal (see Section 4). This subset of MINEMO terms is listed below.

**Subset of MINEMO terms that are required to save data to the NEMO portal (in addition to unique ID for each table).**
1. Research lab (General Features)
  - Institution
  - Principal investigator (PI)
2. Experiment (General features)
  - Experiment paradigm(s)
3. Publication
  - Publication type
  - DOI or File location (Path)
4. Study subjects (Group characteristics)
  - Diagnostic classification
  - Genus
  - Species
  - Age (average)
  - Gender (#male, female subjects)
  - Handedness (#RH, LH subjects)
  - Native language (modal)
5. Experiment condition
  - Experiment condition
  - Experiment task (Instructions)
6. Stimulus presentation
  - Target stimulus type
  - Target stimulus modality
7. Behavioral data collection
  - Response type
  - Response modality
8. EEG Data collection
  - Electrode array (Layout)
  - Sampling rate
9. EEG/ERP Data preprocessing
  - ERP event
  - ERP epoch length (in ms)
  - ERP baseline (pre-target) duration
  - Offline reference
10. EEG/ERP Data file
  - Data file contents (EEG data type)
  - Data file format
  - Data file location (URI)

## MINEMO tools and application

In this section we describe how MINEMO supports our main goal for the NEMO project: to develop methods for cross-lab integration of ERP data. To achieve this goal, it was necessary to annotate data (spatial and temporal metrics) and metadata (data provenance) from ERP experiments using consistent terms.

### The NEMO ontology: Annotation of ERP spatial and temporal metrics

**Figure 1:**
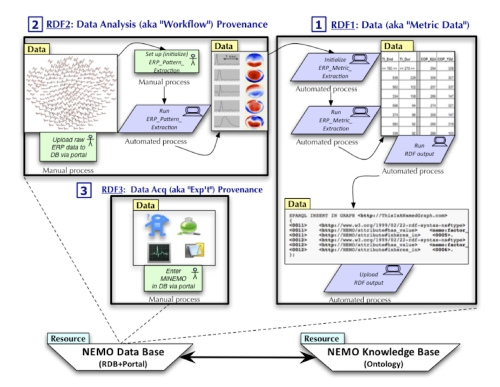
**Box [1]**: Data-driven ERP pattern analysis. **Box [2]**: Generation of spatial and temporal metrics (expressed in RDF). **Box [3]**: Use of NEMO portal for entry of experiment metadata.

ERP data are characteristically described in terms of intensity (in microvolts), distributed over space (electrodes) and time (in milliseconds or samples). To capture spatial and temporal metrics, we use data-driven methods for ERP pattern analysis (Figure 1, Box [1]). and metric generation (Figure 1,Box [2]). The metrics provide a compact summary of ERP patterns and are expressed in RDF (resource description framework) using terms from the NEMO ontology. The metric and RDF generation processes are fully automated.

In addition to spatial and temporal features, which are automatically extracted using the NEMO ERP Toolkit, we capture experiment metadata through the NEMO portal (Figure 1, Box 3; see Section 4.2 for details). Once ERP spatial, temporal, and functional (experimental) features have been expressed in RDF, the NEMO ontology can be used to classify and label the spatiotemporal patterns that are represented by these features (see Refs [1-3] for further details). Thus, ontology-based labeling of data (via RDF) gives a powerful way to link ERP data to a larger base of information that can be used for classification and integration.

### The NEMO portal: Annotation of ERP experiment metadata

The main motivation for MINEMO is to provide a controlled vocabulary for annotation of ERP metadata. In previous work, we showed that both temporal and spatial metrics are needed for accurate classification of ERP data [19,20]. In addition, however, many ERP patterns are also characterized by the functional (i.e., experimental) context in which the data were acquired. For example, the topographic distribution of the well-known N100 pattern is different for visual and auditory stimuli, reflecting activation of distinct neural networks in visual and auditory processing [21]. Similarly, the visual evoked N100 is often greater over the left side of the scalp in response to words, but is bilateral or right-lateralized in response to faces [22].

**Figure 2:**
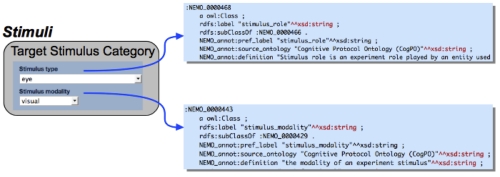
Sample metadata field in NEMO portal and illustration of "tooltips."

Ideally, experiment metadata should be provided when a dataset is submitted for NEMO ERP analysis. To this end, we created a web application (the NEMO portal), database and services that enable NEMO users to record their experiment metadata online through a simple web interface at the same time that they upload their actual datasets to the NEMO database. The NEMO portal [23] is built around three objects: Users, Laboratories, and Experiments. Each user represents an individual researcher and is also a member of some laboratory. In order to access most functions within the portal, a researcher must obtain a user account. Once an account is created, the researcher can login to the portal and start the process of creating an experiment entry. When creating an experiment entry, the researcher enters MINEMO information through a series of HTML forms. The metadata fields correspond with entities in the NEMO ontology; in other words, we capture through the portal a complete description of an experiment, consistent with the standard established by the NEMO ontology and by the MINEMO checklist. To assist portal users, we created a tooltip mechanism that overlays ontology information directly on any form item when the user hovers their mouse pointer over that item. If the user is unsure of the meaning of an item while filling out a form, they can quickly lookup the ontology definition of that item using the tooltip overlay, as depicted in Figure 2.

All form information is saved to an SQL database. Saved experiments can be edited at any time, and previously entered information can be copied and modified for inclusion in new entries, to reduce redundant data entry.

**Figure 3:**
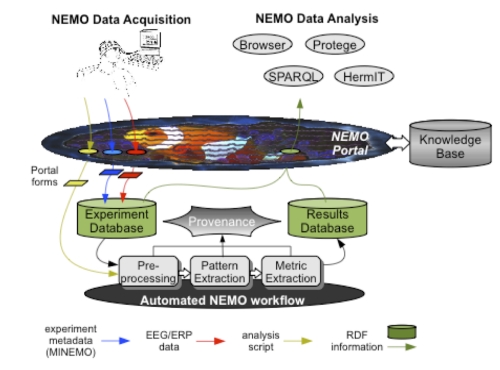
Overview of links between NEMO portal, database, ontology and MI checklist.

Figure 3 gives a conceptual overview of how the NEMO portal and database make contact with the NEMO ontology and MI checklist. Notice that experiment metadata are written out to RDF (Figure 3, bottom right) and are then combined with the RDF representation of spatial and temporal metrics, which are stored in a Results Database.

Once experiment metadata have been captured in RDF, they can then be combined with the spatial and temporal metrics to provide a complete description of ERP patterns for input to classification and meta-analysis.

## Summary and conclusion

### Community participation

NEMO is a relatively new project, and our initial efforts have been focused on developing and testing ERP ontologies and ontology-based tools for analysis. Our next step will be to apply these methods and tools to high-dimensional ERP datasets (with 100 EEG sensors or more) that have been collected across our research sites and to report findings from our first cross-lab, cross-experiment meta-analysis.

Once we have provided this important "proof of concept," we will solicit feedback from the wider clinical and cognitive neuroscience communities. All NEMO ontology (owl) files and NEMO ERP analysis and RDF generation code are freely available from our source forge repository [24]. Documentation is available from our Wiki [25]. We encourage members of the community to browse and download these resources and to provide feedback to our development team. To this end, we have established a public listserv [26].

### Future work

Future work will extend the NEMO portal to support data analysis workflows and to capture workflow provenance in the process. To support this effort, we will adopt parts of two provenance ontologies, the Open Provenance Model (OPM [27]; ) and Provenir ( [28]). The NEMO portal will then be used to store workflow provenance in database structures that are mapped to the NEMO ontology. We think that capturing the context for data acquisition and analysis, and the rich set of parameters that are associated with these processes, will be critically important for accurate comparison of ERP patterns that are the result of different analysis workflows.

### Summary and Conclusion

In conclusion, we have described the development and application of *MINEMO* (Minimal Information for Neural ElectroMagnetic Ontologies), a checklist for description of event-related potentials (ERP) studies. MINEMO extends MINI (Minimal Information for Neuroscience Investigations) to the ERP domain. Checklist terms are explicated in *NEMO*, a formal ontology that is designed to support ERP data sharing and integration. MINEMO is also linked to an ERP database and web application (the *NEMO portal*), which enables the capture of experimental provenance through a direct implementation of MINEMO [29]. Each item on the MINEMO list is encoded in an HTML form on the NEMO Portal and stored in a SQL database. The database also stores metadata entries in RDF (Resource Description Framework), along with summary metrics, i.e., spatial and temporal metadata. Together these spatial, temporal, and functional metadata provide a complete description of ERP data and the context in which these data were acquired. The RDF files then serve as inputs to ontology-based labeling and meta-analysis.

We believe this approach can lead to important new discoveries, for example, by enabling us to compare neural patterns across study paradigms that have distinct but overlapping experimental contexts (e.g., studies of episodic and semantic memory and word comprehension [1]). Given the active investment in similar activities across the sciences, there is a strong possibility that these efforts could lead to knowledge integration, or consilience, across traditional boundaries. The path to this outcome will require dedicated work and collaboration of many groups; the payoff, though, seems well worth the effort.

## Appendix A: MINEMO Checklist

1. Research lab (General features)
  - **Lab ID*
  - **Institution*
  - **Principal investigator (PI)*
  - PI email address
  - PI mailing address
2. Experiment (general features)
  - **Experiment ID*
  - *Experimental paradigm(s)
  - Start date for data collection
  - End date for data collection
3. Publication
  - *Publication ID
  - **Publication type*
  - First author
  - Publication date
  - Title of paper
  - Book or Journal
  - **DOI or File Location (Path)*
4. Study subjects (group characteristics)
  - **Subject group ID*
  - **Diagnostic classification*
  - **Genus*
  - **Species*
  - **Age (average)*
  - **Gender (#male, female subjects)*
  - **Handedness (#RH, LH subjects)*
  - **Native language (modal)*
5. Experiment condition
  - *Condition ID
  - **Experiment condition*
  - **Experiment task (Instructions)*
  - Number of trials per condition
6. Stimulus Presentation
  - *Stimulus type ID
  - Stimulus presentation device
  - Stimulus presentation software
  - **Target stimulus type*
  - **Target stimulus modality*
  - Target stimulus duration
  - Prime stimulus type (if relevant)
  - Prime stimulus modality (if relevant)
  - Prime stimulus duration (if relevant)
  - Prime-Target ISI (if relevant)
  - Prime-Target SOA (if relevant)
7. Behavioral data collection
  - *Response type ID
  - Response collection device
  - Response presentation software
  - **Response type*
  - **Response modality*
  - Response deadline
  - Response accuracy (average)
  - Response time (average)
8. EEG Data Collection
  - Electrode array (Manufacturer)
  - **Electrode array (Layout)*
  - Reference electrode
  - Ground (noise) electrode
  - Scalp-to-Electrode impedance threshold
  - Amplifier gain
  - Amplifier input impedance
  - **Sampling rate*
  - Amplifier filter setting(s)
9. EEG/ERP Data preprocessing
  - Digital filter transformation(s)
  - Digital cleaning method(s)
  - **ERP event*
  - **ERP epochlength (in ms)*
  - **ERP baseline (pre-Target) duration*
  - **Offline reference*
10. EEG/ERP Data file
  - Data file contents (EEG data type)
  - Data file format
  - Data file location (URI)
    * Denotes required field for entry of data in NEMO portal

## Appendix B: MINEMO Term Definitions and Ontology URI

**Table 1:** Research Lab

**Term**	**URI (NEMO)**	**Definition**
Lab ID	NEMO_7431000	A unique identifier for the facility where data were collected
Institution	NEMO_1725000	University or other institution (hospital, company) where data were collected
Principal Investigator (PI)	OBI_0000103	Person responsible for the overall conduct of the study
PI email address	NEMO_8251000	Email address for PI
PI postal address	NEMO_0670000	Postal address for PI

**Table 2:** Experiment (General Features)

**Term**	**URI (NEMO)**	**Definition**
Experiment ID	NEMO_0000537	A unique identifier for the experiment
Start date	NEMO_8539000	Start date for experiment (YYYY)
End date	NEMO_0917000	End date for experiment (YYYY)

**Table 3:** Documentation

**Term**	**URI (NEMO)**	**Definition**
Publication type	BRO: Narrative_Resource	Type of document (journal article, book chapter, unpublished manucript, etc.)
First author	IAO_0000302	First author (family name) on document
Publication date	NEMO_1264000	Date of publication (YYYY)
Title of paper	NEMO_1010000	Title of paper
Title of volume	NEMO_5339000	Title of volume (e.g., journal, book, or conference proceeding)
DOI or File location	NEMO_2062000	Label that denotes the unique location of the document

**Table 4:** Study Subjects (Group Characteristics)

**Term**	**URI (NEMO)**	**Definition**
Subject Group ID	NEMO_2014000	Subject group identifier
Diagnostic Classification	NEMO_5159000	Default classification is "normal"
Genus	NEMO_5621000	Default category is "homo"
Species	NEMO_3454000	Default category is "sapiens"
Age (average)	NEMO_2506000	Average age (in years) of study group
Gender (count)	NEMO_6503000	Number of male, female subjects
Handedness (count)	NEMO_7467000	Number of RH, LH subjects
Native language (modal)	NEMO_5035000	Native language(s) spoken by subjects

**Table 5:** Experiment Context, Paradigm

**Term**	**URI (NEMO)**	**Definition**
Experiment Paradigm	NEMO_0000379	A factorial design (specification of study variables) that is implemented in an experiment (cf. BrainMapLex)
ECI Software	NEMO_1752000	Software application that controls timing of stimulus presentation and recording of behavioral and neural responses
Experiment Condition	NEMO_0000382	A recognizable set of experiment features (stimulus and response type, instructions)
Task (Instructions)	NEMO_0000383	Explicit direction that guides subject behavior during experiment (cf. BrainMapLex)
Number trials (per condition)	NEMO_6697000	The number of trials in an experiment condition

**Table 6:** Stimulus Presentation

**Term**	**URI (NEMO)**	**Definition**
Stimulus presentation device	NEMO_8446000	A device that is used for stimulus presentation
Target stimulus (type)	NEMO_5065000	Role of stimulus that has features which study participants areasked to attend to and/or select for further processing
Target stimulus modality	NEMO_0000443	Modality of target stimulus (visual, auditory, etc.)
Target stimulus duration	NEMO_3331000	Duration of target stimulus (in ms.)
Prime stimulus (type)	NEMO_2367000	Stimulus that precedes the target and is intended to affecttarget processing
Prime stimulus modality	NEMO_0000443	Modality of prime stimulus (visual, auditory, etc.)
Prime stimulus duration	NEMO_5109000	Duration of prime stimulus (in ms.)
Prime–Target ISI	NEMO_8410000	Time interval that separates offset of prime and onset of target stimulus

**Table 7:** Behavioral Data Collection

**Term**	**URI (NEMO)**	**Definition**
Response collection device	NEMO_0000503	A device that is used to record behavioral data
Response type	NEMO_0000467	A behavioral process that occurs an experimentin response to stimulus
Response modality	NEMO_0000756	Class of body parts used to perform actions that can play the role of a response (cogPO)
Response deadline	NEMO_2473000	The maximum time that is allowed for an experiment response
Accuracy (average)	NEMO_0000431	The accuracy (correctness) of an experimental response
Response time (average)	NEMO_0000433	The latency (onset time) of an experimental response

**Table 8:** EEG/ERP Data Collection

**Term**	**URI (NEMO)**	**Definition**
Electrode array (manufacturer)	NEMO_3240000	Name of company that produced EEG sensors and sensor array
Electrode array (layout)	NEMO_6227000	Number of EEG sensors and topographic layout of sensors
Recording reference	NEMO_6771000	10-20 location of electrode(s) used as EEG recording reference
Ground (noise) electrode	NEMO_4335000	10-20 location of electrode(s) used as isolated common (aka "ground")
Scalp-to-electrode impedance threshold	NEMO_6541000	Maximum scalp-to-electrode impedance (in Hz)
Amplifier gain		Resolution (in bits/microvolt)
Amplifier input impedance	NEMO_2655000	Input impedance to amp (in Hz)
Temporal sampling rate	NEMO_2585000	Sampling rate (in ms)
Amplifier filter setting	NEMO_1142000	Analog bandpass (in Hz)

**Table 9:** EEG/ERP Data File

**Term**	**URI (NEMO)**	**Definition**
Data file contents	NEMO_2662000	Type of data (continuous, segmented, or averaged EEG)
Data file format	NEMO_1194000	Data file format (.raw, .txt, etc.)
Data file location	NEMO_3087000	Unique resource identifier or file path

**Table 10:** EEG/ERP Data Preprocessing

**Term**	**URI (NEMO)**	**Definition**
Digital filter transformation	NEMO_7669000	Offline removal of signal above or below a certain frequency level
Data cleaning transformation	NEMO_4273000	Offline removal of any signal that is not of interest to the researcher
ERP event (for averaging)	NEMO_6783000	The role ofan event (e.g., stimulus onset)that is used forEEG averaging
ERP epoch length	NEMO_3620000	The durationof an ERP, where the time of the event is designated as time zero.
ERP baseline length	NEMO_6232000	The durationof ERP baseline (by default, the end of the baseline is the onset of the ERP)
Offline reference	NEMO_0000321	Offline schema used for re-reference

^1^ An alternative to the use of a controlled vocabulary is to discover mappings between data annotations [19,20]. However, this process is nontrivial, and its success depends on the nature and amount of variability in the data.
